# Low dose of dexmedetomidine as an adjuvant to bupivacaine in cesarean surgery provides better intraoperative somato-visceral sensory block characteristics and postoperative analgesia

**DOI:** 10.18632/oncotarget.18864

**Published:** 2017-06-29

**Authors:** Yong-Hong Bi, Xiao-Guang Cui, Rui-Qin Zhang, Chun-Yu Song, Yan-Zhuo Zhang

**Affiliations:** ^1^ Department of Anesthesiology, China and Heilongjiang Key Laboratory for Anesthesia and Critical Care, The Second Affiliated Hospital of Harbin Medical University, Harbin, China

**Keywords:** dexmedetomidine, cesarean surgery, somato-visceral sensory block, postoperative analgesia

## Abstract

**Object:**

In this study, we aimed to investigate the beneficial effects of dexmedetomidine on somato-visceral sensory block characteristcs, postoperative analgesia and stress response of intrathecal bupivacaine administration in women undergoing cesarean section, and to find out which dose is better.

**Methods:**

Sixty parturients with the American Society of Anesthesiologists (ASA) physical status I or II were anesthetized with intrathecal bupivacaine(10mg) alone or in combination with dexmedetomidine (3 μg and 5 μg) to undergo cesarean section. The anesthetic parameters, postoperative analgesia and stress responses were monitored.

**Results:**

Co-administration of dexmedetomidine(3 μg and 5 μg) prolonged the duration of motor and sensory block compared with bupivacaine(10mg) alone. Less supplemental dose of lidocaine and fentanyl were required in dexmedetomidine(3 μg and 5 μg) co-administration groups. Visceral traction response and abdominal muscle relaxation in operation were better in dexmedetomidine(3 μg and 5 μg) co-administration groups. No difference in haemodynamics was detected among groups. There was no significant difference in Apgar scores, neonatal umbilical pH, oxygen pressure, carbon dioxide pressure and lactate level among groups. Postoperative plasma IL-6 and cortisol levels were lower in dexmedetomidine(3 μg and 5 μg) co-administration groups. At 6 hour after operation the visual analogue scale (VAS) was smaller in dexmedetomidine(3 μg and 5 μg) co-administration groups. The uterine contraction pain at 6 and 12 hour after operation and supplemental analgesics had no difference across three groups. No difference of side effects(shivering, nausea and vomiting, itching), the first anal aerofluxus time and intraoperation tramadol dose were detected among the three groups.

**Conclusion:**

The use of dexmedetomidine especially at the dose of 3μg as an adjuvant to bupivacaine in cesarean surgery provides better intraoperative somato-visceral sensory block characteristcs and postoperative analgesia, which produced no influence on Apgar scores, side effects and stress response.

## INTRODUCTION

Spinal anesthesia is commonly used in cesarean section surgery. Apart from being economical and easy to administer, spinal anesthesia provides both analgesia and muscular relaxation with rapid onset of action [1]. However, the administration of local anesthetics alone has a short duration of effect, and is insufficient for preventing visceral pain and nausea especially at an earlier stage [2–4]. Visceral pain is common during spinal anesthesia with mini dose local anesthetics. It is especially uncomfortable in cesarean surgery as the surgeons need to lift the uterus and suture the peritoneal during surgery. Moreover, there remains a lack of long lasting postoperative analgesia [5]. To overcome the defects of local anesthetics, joint administration of adjuvant drugs has become an widely accepted practice in clinical work.

Adjuvant drugs added to the intrathecal bupivacaine can decrease the dose of local anesthetics and guarantee sensory and motor block. Intrathecal adjuvants include opioids, agonist, magnesium, neostigmine, ketamine and midazolam etc. Clonidine and dexmedetomidine are receptor agonists which have sedative, analgesic, perioperative sympatholytic, anesthetic-sparing, and hemodynamic-stabilizing properties [6]. Clonidine provides a dose-dependent increase in the duration of sensory and motor block, besides antinociceptive properties [7]. Furthermore, evidence from animal study indicates that dexmedetomidine produces spinal analgesia as efficiently as clonidine [8]. Intrathecal α_2_-receptor agonists are found to have antinociceptive action for both somatic and visceral pain [9]. Intrathecal dexmedetomidine has been used in the dose of 3, 5, 10 and 15 μg along with bupivacaine in surgeries such as lower limbs, transurethral prostatectomy [9–14]. Intrathecal dexmedetomidine has also been used in cesarean section. Sun Y et al. demonstrated that addition of 10 μg dexmedetomidine into bupivacaine provided better intraoperative and postoperative analgesia [15]. Li Z et al. showed the same data that dexmedetomidine at the dose of 10μg adjunt to bupivacaine is sufficient to provide adequate anesthesia and postoperative analgesia [16]. It remains unknown whether addition of lower doses of dexmedetomidine to bupivacaine could produce satisfactory decrease of visceral pain in cesarean surgery. Therefore, in the current study we aimed to test whether adjunct use of lower doses (3 and 5 μg) of dexmedetomidine with bupivacaine intrathecally could improve somato-visceral block characteristcs and decrease postoperative pain without affecting the infants.

## RESULTS

### Demographic and surgical characteristics

The demographic profiles of the patients in all the 3 groups were comparable with regard to age, weight, height, gestation age, mean duration of surgery (P > 0.05). There were no significant difference between duration of surgery, surgery starting time and fetal delivery time among groups (Table 1).

**Table 1:** Demographic and surgical characteristics

	Bup	Bup+Dex(3)	Bup+Dex(5)	F/χ^2^	*P* value
Age (year)	29.5±3.9	32.1±4.9	31.3±3.7	2.04	0.1398
Height(cm)	161.2±5.4	161.2±3.6	162.0±3.7	0.23	0.7944
Weight(kg)	74.9±8.4	75.9±7.3	72.9±9.6	0.65	0.5266
Gestational weeks	38.5(37.0,39.0)	39.0(38.0,39.0)	39.0(38.0,40.0)	4.51	0.1051
Duration of surgery(min)	48.0±8.8	47.3±11.5	53.8±12.7	1.91	0.1570
Onset time of operation (min)	13.1±3.4	14.7±3.8	13.6±3.1	0.71	0.4960
Fetal delivery time(min)	19.6±20.0	23.1±6.9	21.9±4.1	2.42	0.0981

### Spinal block characteristics and analgesia

The spinal block characteristics are presented in Table 2. The cases with supplemental lidocaine reduced to 7 and 5 in Bup+Dex(3) and Bup+Dex(5) groups (p<0.05), respectively. The supplemental lidocaine dose was higher in Bup group than Bup+Dex(3) and Bup+Dex(5) groups (p<0.05 vs Bup). The cases with visceral pain and received fentanyl administration were 9 in Bup group, 2 in Bup+Dex(3) group and 6 in Bup+Dex(5) group (p<0.05 vs Bup). Fentanyl dose was higher in Bup group than the other two groups. The abdominal muscle relax satisfactory cases were 7 in Bup group, 15 in Bup+Dex(3) group and 16 in Bup+Dex(5) group. The time to highest sensory block was similar among the 3 groups. Duration of motor block was significantly prolonged from 3.56±1.02h in Bup group to 5.82±0.95h in Bup+Dex(3) group and 5.14±0.88h Bup+Dex(5) group. The other spinal block characteristics including highest sensory block level among groups.

**Table 2:** Spinal block characteristics

	Bup	Bup+Dex(3)	Bup+Dex(5)	F/χ^2^	*P* value
Time to Max sensory level(min)	15.0(10.0,15.0)	15.0(12.5,20.5)	15.0(10.0,17.5)	1.21	0.5468
Supplemental lidocaine cases	14(70.0)^ab^	7(35.0)^a^	5(25.0)^b^	9.10	0.0110
Supplemental 2%lidocaine dose(ml)	5.0(2.5,10.0)^ab^	5.0(0.0,11.0)^a^	2.5(0.0,5.0)^b^	11.58	0.0031
Fentanyl cases(%)	9(45.0)^ab^	2(10.0)^a^	6(30.0)^b^	6.07	0.0480
Fentanyl dose(mg)	0.0(0.0,0.1)^ab^	0.0(0.0,0.0)^a^	0.0(0.0,0.05)^b^	6.69	0.0352
Visceral pain cases(%)	9(45.0)^ab^	2(10.0)^a^	6(30.0)^b^	6.07	0.0480
Visceral pain time(min)	37.0(35.0,40.0)	38.5(37.0,40.0)	42.5(40.0,45.0)	5.27	0.0716
Muscle relaxation satisfaction cases(%)	7(35.0)^ab^	15(75.0)^a^	16(80.0)^b^	10.48	0.0053
Duration of motor block(h)	3.6±1.0^ab^	5.8±1.0^a^	5.1±0.9^b^	29.88	<0.0001

a,b suggest groups labeled by the same letter have statistical significance. Data in accordance with normal distribution were showed by mean ± standard deviation. Data not in accordance with normal distribution were showed by median.

The VAS at 6h after surgery was higher in Bup group than in Bup+Dex(3) and Bup+Dex(5) groups (Table 3). No difference of VAS was observed at 12h after surgery. There was no difference in uterine contraction pain after surgery and supplement postoperative analgesia time (Table 3).

**Table 3:** Postoperative pain and analgesia

	Bup n (%)	Bup+Dex(3) n (%)	Bup+Dex(5) n (%)	F/χ^2^	*P* value
VAS					
6h	0.0(1.5,2.5)^ab^	0.0(0.0,0.0)^a^	0.0(0.0,1.5)^b^	11.50	0.0032
12h	4.0(5.5,6.5)	5.0(2.5,7.5)	3.8(2.5,5.3)	2.08	0.3533
Uterine Contraction Pain					
6h	0.0(0.0,0.0)	0.0(0.0,0.0)	0.0(0.0,0.0)	1.72	0.4239
12h	1.0(1.0,1.0)	1.0(0.5,1.0)	1.0(0.5,1.0)	1.84	0.3978
Supplement postoperative analgesia case(%)	5(25.0)	4(20.0)	8(40.0)	2.13	0.3440
Postoperative supplement drug time(h)	22.3±3.4	24.8±3.0	23.8±5.4	0.35	0.7096
Postoperative promethazine	0.0(0.0,0.0)	0.0(0.0,0.0)	0.0(0.0,0.0)	0.00	1.0000

a,b suggest groups labeled by the same letter have statistical significance. Data in accordance with normal distribution were showed by mean ± standard deviation. Data not in accordance with normal distribution were showed by median.

### Fetal characteristcs

In all three groups newborns have no signs of fetal distress, evidenced by Apgar score 9 and 10 at 1 and 5 min, respectively (Table 4). There was no difference in umbilical oxygen partial pressure, dioxide partial pressure, glucose and lactate among three groups (Table 4).

**Table 4:** Apgar scores and umbilical artery gas analysis

Factor	Bup	Bup+Dex(3)	Bup+Dex(5)	F	*P* value
Apgar score					
1min	9.0(8.0,9.0)	9.0(8.0,9.0)	9.0(8.0,9.0)	0.59	0.7450
5min	10.0(9.0,10.0)	10.0(9.0,10.0)	10.0(9.5,10.0)	0.31	0.8556
Umbilical oxygen partial pressure(mmHg)	24.8±10.3	25.7±8.4	27.6±9.0	0.48	0.6217
Umbilical dioxide partial pressure(mmHg)	41.9±5.1	43.1±5.4	43.6±5.9	0.49	0.6162
Umbilical glucose(mmol/L)	3.5±0.6	3.6±0.4	3.6±0.6	0.12	0.8838
Umbilical lactate(mmol/L)	1.5(1.4,1.6)	1.5(1.3,1.7)	1.4(1.2,1.6)	1.33	0.5136
Umbilical blood PH	7.4±0.0	7.3±0.0	7.3±0.0	1.99	0.1460

### Hemodynamics and side effects

**Figure 1:**
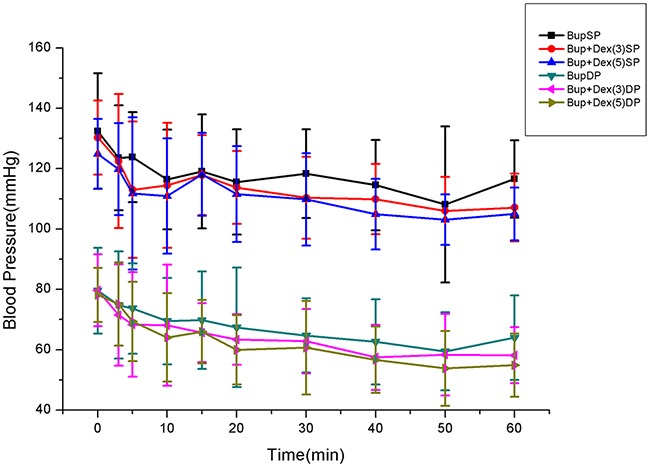
Comparison of SP, DP among groups

**Figure 2:**
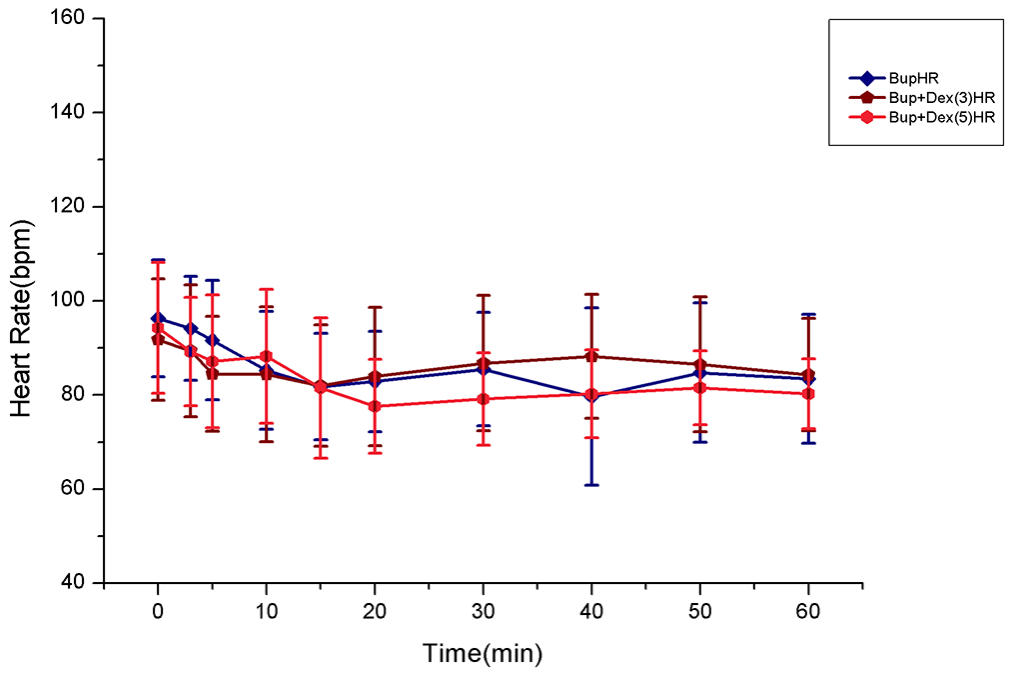
Comparison of HR among groups

There was no difference among three groups in SP, DP and HR (Figures 1 and 2).

The phenylephrine dose at 10min after spinal anesthesia was 190.0±88.4 μg in Bup+Dex(5), which is higher than in Bup group 133.0±39.1 μg and Bup+Dex(3) group 130.0±43.3μg (Table 5). The phenylephrine dose at 20min after spinal anesthesia was 308.0±111.5μg in Bup+Dex(5) group, 229.5±59.6 μg in Bup group and 241.0±64.1 μg in Bup+Dex(3) group (P<0.05 vs Bup+Dex(5)). No difference was observed between Bup and Bup+Dex(3) groups (Table 5). There were no significant difference between nausea and vomiting intraoperation, postoperation, shiver, pruritus, exaust time and tramadol dose among groups (Table 6).

**Table 5:** Phenylephrine dose

Factor	Bup	Bup+Dex(3)	Bup+Dex(5)	F	*P* value
Phenylephrine dose(μg)					
10min	133.0±39.1^a^	130.0±43.3^b^	190.0±88.4^ab^	6.12	0.0039
20min	229.5±59.6^a^	241.0±64.1^b^	308.0±111.5^ab^	5.37	0.0073

a, b suggest groups labeled by the same letter have statistical significance.

**Table 6:** Comparison of incidence of side effects, first anal aerofluxus time and tramadol dose

	Bup n(%)	Bup+Dex(3) n(%)	Bup+Dex(5) n(%)	F/χ^2^	*P* value
Nausea and vomiting intraoperation	0(0.0)	4(20.0)	3(15.0)	4.47	0.1442
Nausea and vomiting postoperation	0(0.0)	1(5.0)	1(5.0)	1.28	1.0000
Shivering postoperation	11(55.0)	8(40.0)	9(45.0)	0.94	0.6258
Pruritus postoperation	2(10.0)	3(15.0)	3(15.0)	0.42	1.0000
First anal aerofluxus time(h)	35.5±9.9	35.4±9.1	34.8±6.7	0.04	0.9644
Tramadol case intraoperation	3(15.0)	3(15.0)	1(5.0)	1.24	0.5375

### Stress response

There was no difference in the maternal baseline IL-6 level among groups. The postoperative IL-6 level was lower in Bup+Dex(3) and Bup+Dex(5) groups than Bup group (Table 7). Baseline maternal cortisol levels were similar among Bup, Bup+Dex(3), and Bup+Dex(5) groups (87.6±10.3μg/L, 86.8±10.0μg/L, 93.4±9.5μg/L, respectively). After surgery, cortisol level was lower in Bup+Dex(3) and Bup+Dex(5) groups than Bup group (Table 7).

**Table 7:** Compare the stress response effect

	Bup	Bup+Dex(3)	Bup+Dex(5)	F/χ^2^	*P* value
Preoperative IL-6 (μg/L)	50.8±7.2	50.4±6.5	50.9±4.9	0.03	0.9711
Postoperative IL-6 (μg/L)	107.3±6.2^ab^	87.3±4.7^a^	88.2±6.1^b^	78.61	<0.0001
Preoperative cortisol (μg/L)	87.6±10.3	86.8±10.0	93.4±9.5	2.67	0.0778
Postoperative cortisol (μg/L)	157.7±18.4^ab^	124.7±11.1^a^	128.1±7.4^b^	38.18	<0.0001

a, b suggest groups labeled by the same letter have statistical significance.

## DISCUSSION

The selection of different combination and suitable doses when using adjuvant with local anesthetics is a critical process and signifies the consideration of factors such as the formation and duration of sensory and motor block, the quality and duration of postoperative analgesia, and the side effects that might be observed in the mothers and the newborns [17]. Over the years, many drugs have been used intrathecally as an adjuvant to local anesthetic to prolong the intraoperative as well as postoperative analgesia with variable effects [18].

Dexmedetomidine is a new and more selective α_2_ receptor agonist compared to clonidine, with higher sedative and analgesic effects. Dexmedetomidine provides stable hemodynamic conditions, good sedation, and good quality of intraoperative and prolonged postoperative analgesia with minimal side effects [19]. Our study indicates that in comparison to 5μg intrathecal dexmedetomidine, 3μg dexmedetomidine prolonged and intensified sensory and motor block of bupivacaine without causing any significant side effects. Dexmedetomidine prolonged the pain free period and improved postoperative analgesia. The results are in consistent with previous findings [9, 10], which indicated that the addition of dexmedetomidine demonstrated effective spinal block. The difference is that the dose employed in this study is lower than previous ones. The reason for prolongation of spinal anesthesia in case of dexmedetomidine is due to its supra-spinal action at locus ceruleus and dorsal raphe nucleus. Moreover, dexmedetomidine is more selective to α_2_ receptor than clonidine, with more sedative and analgesic effects. The prolongation of the motor block of dexmedetomidine with bupivacaine can be explained by the binding of agonists to motor neurons in the dorsal horn [20] and the synergism between local anesthetic and agonists [21].

Hypotension is very common in neuroaxial blocks for cesarean section. This is particularly due to sympathetic block and tends to be treated with ephedrine, phenylephrine and crystalloid-colloid solution infusion [22, 23]. In this study, addition of 5μg dexmedetomidine to hyperbaric bupivacaine produced unstable hemodynamics after spinal anesthesia. However, low dose dexmedetomidine(3μg) adding into bupivacaine exerted stable hemodynamics. Addition of 5μg dexmedetomidine caused lower systolic pressure and diastolic pressure than bupivacaine alone or addition of 3μg dexmedetomidine at all time after spinal anesthesia. Therefore, more phenylephrine(190.0±88.3μg) was administered to maintain blood pressure in patients giving adjuvant 5μg dexmedetomidine than bupivacaine alone (133.0±39.1μg) or addition of 3μg dexmedetomidine (130.0±41.3μg) groups at 10 min after spinal anesthesia. These data indicated that low dose dexmedetomidine(3μg) added into hyperbaric bupivacaine could prolong its sensory and motor block effect with stable blood pressure. The benefits of the use of minidose dexmedetomidine is that it provides prolonged postoperative analgesia and avoided hemodynamic instability such as hypotension and bradycardia produced by larger dose of dexmedetomidine or a prolonged motor blockade.

Several studies have shown blunting of the cardiovascular responses to operation, surgical stimulation and extubation with the use of dexmedetomidine for abdominal hysterectomy on the stress response during caesarean delivery [24]. Nasr and Abdelhamid researched the effect of caudal dexmedetomidine versus fentanyl and bupivacaine on the stress response and postoperative analgesia in pediatric cardiac surgery, and found that dexmetomidine attenuated the stress response and produced better analgesia [25].

Kang et al. reported that dexmedetomidine administration during surgery reduced intraoperative and post-operative secretion of cytokines, including the pro-inflammatory cytokines tumour necrosis factor-α, interleukin-1β and IL-6 and anti-inflammatory cytokines IL-4 and CRP level in their study [26]. Nour EM et al. also found that epidural administration of dexmedetomidine adjunct to bupivacaine inhibited the increment of plasma interleukin-6 [27]. Similarly, in this study the postoperative IL-6 level was lower in Bup+Dex(3) and Bup+Dex(5) groups than Bup group in our study. Preoperative anxiety, fear, sleeplessness, anesthesia, surgery and postoperative pain could elevate cortisol level which is the terminal hormone of pituitary-adrenal cortex axis dramatically. In our study we found that cortisol secretion was suppressed by low dose dexmedetomidine.

Dexmedetomidine and clonidine prevent postoperative shivering by inhibiting central thermoregulation and attenuation of hyperadrenergic response to perioperative stress [28]. Previous studies reported that the incidence of shivering is 10-30 % in control group and there is no occurrence of shivering in dexmedetomidine group [29, 30]. In our study, shivering occurred in 40% and 45% of the patients in the dexmedetomidine(3μg) and dexmedetomidine(5μg) groups, and 55% in control group. The reasons for the difference may be that the small dose of dexmedetomidine (3μg and 5μg) given intrathecally is not as effective as intravenous application to prevent shivering, and parturients are prone to lose more heat and blood than other surgery.

In all three groups, the newborns have no signs of fetal distress, evidenced by Apgar score 9 and 10 at 1 and 5 min, respectively, which infers the advantageous use of dexmedetomidine over other adjuvants. The results were parallel to those reported in literature [15, 31]. Furthermore in our study on significant difference in the incidence of side effects such as pruritus, nausea and vomiting were noted across the three groups. Similar results were reported in previous studies [10, 32, 33]. There has been much debate regarding problems with breastfeeding after anesthesia. Unfortunately, there are no published studies on the safety of breastfeeding after epidural dexmedetomidine when used as an adjunct in labor analgesia. According to the data of previous study [34], we recommended that breastfeeding should be avoided during the 24h immediately after surgery.

In summary, the use of low dose of dexmedetomidine (3μg) as an adjuvant to bupivacaine in cesarean surgery provides better intraoperative somato-visceral block characteristcs and postoperative analgesia without significant impact on Apgar scores or incidence of side effects and decreases stress response level.

## MATERIALS AND METHODS

### Design

We designed a prospective, randomized, double-blind study to determine whether intrathecal bupivacaine with dexmedetomidine could improve block characteristcs and decrease stress response for cesarean section, and to find out the minimal dose of dexmedetomidine for parturients.

### Subjects and setting

The study was approved by the institutional ethics committee with written informed consent (the ethics number:ChiCTR-IIR-16008497. Sixty parturients at the age 18-40 years old with American Society of Anesthesiologists (ASA) physical status I or II undergoing elective cesarean section were enrolled in this study. Exclusion criteria included a long history of opioid analgesic use or NSAIDS, psychiatric disorders, preoperative heart rate less than 50 bpm with cardiac conduction or rhythm abnormalities, neuromuscular and endocrine diseases or allergic reactions to α_2-_adrenergic agonist.

### Study protocol

An 18-gauge intravenous cannula was inserted into a peripheral vein. Standard intraoperative monitoring was used, consisting of ECG, pulse oximetry and non-invasive arterial blood pressure. An intravenous infusion of Lactated Ringer's Solution 500ml was administered. Lumber epidural anesthesia was induced with 18 gauge Tuohy needle with parturients in lateral position in lumber 3 -4 or lumber 2-3 interspace. Location of epidural space was confirmed by loss of resistance techniques. The spinal injection was performed with a 25 gauge pencil point needle. A computer-generated randomization table was used to divide parturients into three groups: intrathecal 10mg bupivacaine alone(Bup group), 10mg bupivacaine with 3μg dexmedetomidine (Bup+Dex(3) group), 10mg bupivacaine with 5μg dexmedetomidine (Bup+Dex(5) group). All solutions were at room temperature and diluted with 0.9% saline to a final volume of 2.0 ml. Study drugs were injected at a rate of 1ml/15s by the same anesthesiologist. Epidural catheter was secured 3-5 cm into the epidural space, then parturients were placed supine with a Crawford wedge displacing the uterus to the left until birth. The allocation to one of three combinations was done by a computer-generated randomization scheme. The prescriptions of the study medication were kept in sealed numbered envelopes and stored near the operation room. A registered anesthetic nurse who was not involved in the study prepared the solutions, using the consecutive envelopes, a few minutes before starting the procedure. All employees contributing to the study were blinded for the spinal medication. In cases where sensory block did not reached T_6_ within 20 min after the injection, a general anesthetic was administered. If spinal anesthesia failed, the patients were given epidural drugs, and be excluded from this study. If the patients experienced any discomfort such as back pain, stomach discomfort after the fetal delivery, intravenous fentanyl 0.05mg would be given immediately. If not relieved, another fentanyl 0.05mg would be given. If the patients felt pain around surgery area or the surgeons felt abdominal muscle relaxant not enough, 2% lidocaine 5ml would be given through epidural catheter. If not effective, 2% lidocaine 5ml would be given after 5min. Rescue lidocaine dose with time and fentanyl total dose would be recorded. When the surgeon closed peritoneum, morphine 2.5mg would be given by epidural route as postoperative analgesia. Blood collection finished before anesthesia and after operation. If shivering happened, tramadol 100mg were given intraoperation.

If the patient's VAS was more than 4 after surgery, the surgeon would give them diclofenac sodium and lidocaine hydrochloride injection. If the patient was shivering later on postoperatively, promethazine 12.5mg would be given.

### Measurements

Sensory block was evaluated every 5 min with a pinprick test. Motor block was evaluated with the Bromage scale (0 = no motor loss, 1 = inability to flex the hip, 2 = inability to flex the knee, and 3 = inability to flex the ankle). The following parameters were observed immediately after the administration of spinal block: Maximum sensory level, time to maximum sensory level, duration of motor block (two lower limbs bromage score return to 0), the onset time of operation, fetal delivery time, supplemental lidocaine dose and time intraoperation, supplemental fentanyl dose, visceral pain, abdominal muscle relaxation, patients VAS 6, 12 hour after surgery, first rescue analgesia drug time and the first anal aerofluxus time (the first anal aerofluxus time, anus exhausting time. It reflected the recovery time of gastrointestinal function recovery.). Side effects include shivering, nausea and vomiting, hypotension, pruritus etc.

The hemodynamic parameters include systolic pressure(SP), diastolic pressure (DP), heart rate (HR), the saturation of pulse oximetry at 0, 3, 5, 10, 15, 20, 30, 40, 50, 60 minutes after spinal anesthesia, and dose of phenylephrine at 10, 20 minutes after the spinal anesthesia. If the systolic pressure decreases 20%, or less than 100mmHg, phenylepherine 60-80μg would be given. Umbilical blood gas analysis includes fetal blood pH, oxygen partial pressure, carbon dioxide partial pressure, lactate and Apgar scores.

### Statistic analysis

Data are expressed as mean±SD. Statistical analysis was performed using SAS9.1. ANOVA and χ^2^ test were used for analysis of the standard characteristics, the degree of motor block, sensory block level, maternal side effects, fetal delivery and postoperative analgesia. Analysis of variance for repeated data was used to compare hemodynamic characteristics. P-value less than 0.05 was considered statistically significant for all comparisons.
